# Mesenchymal stem cell-derived exosome subpopulations remained consistent for 28 culture days, displaying therapeutic effects in a silicosis mouse model

**DOI:** 10.3389/fcell.2025.1550447

**Published:** 2025-05-27

**Authors:** Lina Zhang, Jing Jin, Liguang Sun, Gang Hou, Mingming Deng, Yiding Bian, Jianming Liu, Wei Cheng, Shaoliang Xing, Wenjia Wang, Xin Dong, Qingjie Fan, Lei Gao, Xinhua Lei, Yongli Bao, Yongguang Yang

**Affiliations:** ^1^ School of Life Sciences, Northeast Normal University, Changchun, Jilin, China; ^2^ National-Local Joint Engineering Laboratory of Animal Models for Human Disease, The First Hospital of Jilin University, Changchun, Jilin, China; ^3^ Research and Development Department (R&D), Beijing Jizhongke Biotechnology Co., LTD., Beijing, China; ^4^ Department of Pulmonary and Critical Care Medicine, Center of Respiratory Medicine, China-Japan Friendship Hospital, Beijing, China; ^5^ Department of Otolaryngology Head and Neck Surgery, China-Japan Union Hospital, Jilin University, Changchun, Jilin, China; ^6^ Research and Development Department (R&D), Jilin Zhong Ke Bio-engineering Co., LTD., Changchun, Jilin, China

**Keywords:** mesenchymal stem cells exosomes, exosome subpopulation heterogeneity, scalable production, biomanufacturing workflow, natural exosome therapy

## Abstract

**Introduction:**

The clinical translation of mesenchymal stem cell-derived exosome faces critical challenges in scalable production, subpopulation stability, and therapeutic route optimization. This study systematically addresses these barriers to advance exosome-based therapies.

**Methods:**

We established a 28-day biomanufacturing workflow using a Hollow Fiber 3D bioreactor integrated with the RoosterBio exosome-harvesting system. Exosomes were subsequently purified and rigorously characterized at multiple production stages, followed by isotopically labeled with ^89^Zr for biodistribution studies. Therapeutic efficacy was evaluated in a silica-induced mouse silicosis model comparing intravenous and respiratory administration routes.

**Results:**

Our findings indicate that (1) the RoosterBio exosome harvesting system in the Hollow Fiber 3D bioreactor enables 28 days production of exosomes, with stable harvesting of the main subpopulations over a certain period; (2) systemic administration via intravenous injection in rats reveals distinct tissue tropism, with isotope-labeled exosomes exhibiting predominant hepatic accumulation; and (3) in the silica-induced mouse silicosis model, respiratory delivery of exosomes significantly improves disease progression, whereas intravenous infusion of exosomes does not yield notable therapeutic effects.

**Discussion:**

This study proposes a holistic workflow for early-stage development of natural exosomes as therapeutics, offering guidance on industrial-scale production, purification, and characterization of exosomes with stable subpopulation distribution and functional consistency. It further addresses administration route selection in pulmonary disease animal models and heterogeneity assessment of natural exosomes. These advancements facilitate clinical translation of exosome-based therapies.

## 1 Introduction

In modern medical research, mesenchymal stem cells (MSCs) serve as representatives of cell therapy. MSCs possess robust paracrine functions, constituting the main mechanism of their therapeutic effects. They regulate the microenvironment by secreting a range of cytokines, chemical molecules, and growth factors, thereby activating endogenous stem cells for tissue repair after injury (Jovic et al., 2022; Han et al., 2022; Vilar et al., 2023; Bou-Ghannam et al., 2021). MSCs, with such advantages, are the preferred cells for cell therapy and regenerative medicine, used to treat various types of diseases (Uccelli et al., 2008; Pittenger et al., 2019; Hoang et al., 2022). However, clinical applications of MSCs still face many challenges, such as cell senescence, potential tumor formation, low engraftment into target tissues, and inability to cross the blood-brain barrier (Zhou et al., 2021; Ruppert et al., 2018; Musiał-Wysocka, Kot, and Majka, 2019).

Publicized experimental results indicate that the therapeutic effects of MSCs often fall short of expectations. MSC-derived exosomes circumvent these limitations and have emerged as a promising new cell-free therapy. Abundant *in vivo* and *in vitro* studies demonstrate that exosome-based therapies bypass some tricky issues of cell therapy, such as stress-induced necrosis or abnormal differentiation and immune rejection caused by cell transplantation. Moreover, exosomes derived from MSCs have functions similar to parent cells, possessing immunomodulatory, pro-angiogenic, and tissue regenerative abilities (Kou et al., 2022; Soler-Botija et al., 2022; Gowen et al., 2020). For instance, MSC-derived exosomes can reduce the scope of myocardial injury (Fu et al., 2020), promote tissue damage repair (Ding et al., 2021), including acute renal tubular injury (Cao et al., 2021), retinal nerve injury (Mead and Tomarev, 2017), and lung injury (Xiao et al., 2020), as well as facilitate angiogenesis (Heo and Kim, 2022), and modulate the immune system (Zheng et al., 2021).

The main advantages of exosomes as therapeutic nanobiologics can be summarized as follows. Firstly, exosomes serve as mediators of stem cell paracrine actions, participating in intercellular communication and considered the main mechanism of disease treatment (Théry et al., 2002). Secondly, exosomes can be combined with existing or newly developed compounds or methods, designed as carrier particles containing specific components. Targeted delivery of therapeutic molecules to specific cells or tissues can be achieved through surface engineering of exosomes and addition of specific contents into vesicles (Herrmann et al., 2021; Jeppesen et al., 2023; Shao et al., 2023). Lastly, natural exosomes possess autonomous targeting capabilities, evade the reticuloendothelial system (RES) to avoid immune detection, naturally penetrate tissues with dense inflammation, and home to lesion tissues, all of which are conducive to constructing them as drug carriers (Escudé Martinez de Castilla et al., 2021; Gatti et al., 2011). All these characteristics make exosomes an ideal natural material for the development of nanomedicine. Compared to cell therapy, exosome therapy is safer, devoid of potential tumorigenicity of stem cells, and is the optimal choice for cell-free therapy (Xu et al., 2020; Wan et al., 2022; Ha et al., 2020).

Exosomes, once considered a homogeneous population, are now recognized as heterogeneous vesicles with distinct subpopulations, each possessing unique compositions and functions. Studies show that extracellular vesicles (EVs) derived from MSCs vary in biophysical properties, proteomic profiles, and RNA content (Willis et al., 2017; Feng et al., 2024). For instance, Kowal et al. isolated exosome subpopulations based on size using sequential centrifugation and identified distinct tetraspanin-enriched subpopulations (Kowal et al., 2016). Similarly, Lai et al. found that MSC-secreted vesicles vary in RNA and protein content (Lai et al., 2016). These findings suggest that different exosome subpopulations contribute to diverse effects on target cells. A study on bone marrow MSC-derived exosome subpopulations in a bronchopulmonary dysplasia model demonstrated that specific subpopulations mediate therapeutic effects, highlighting the importance of considering exosome subpopulation stability in therapeutic applications (Willms et al., 2016). Therefore, when preparing exosomes for therapy, it is essential to account for the stability of subpopulations during production to define an optimal collection window, ensuring consistent bioactive components and functionality.

Currently, the balance between the cost, yield, purity, activity, and heterogeneity of large-scale MSC-derived exosome production is the main factor limiting the clinical application of exosomes. Various factors such as cell quality, selection of bioreactors, cell expansion culture, differences in exosome secretion systems, long-term storage and transportation, and different exosome purification processes greatly influence the yield, purity, heterogeneity, and activity of exosomes (Gowen et al., 2020; Ahn et al., 2022; Kimiz-Gebologlu and Oncel, 2022; Wang et al., 2023; Kalluri and LeBleu, 2020; Jeyaram and Jay, 2018). Therefore, it is necessary to amplify a large number of cells in 3D bioreactors to produce sufficient exosomes for *in vitro* experiments, *in vivo* animal model testing, and clinical trials (Thippabhotla et al., 2019; Zhang et al., 2021; Lee et al., 2023), as well as to establish a comprehensive nanoparticle characterization platform to control the quality of the harvested liquid at each stage of exosome preparation, to achieve stability and consistency verification after process scaling (Patel et al., 2019; Pan et al., 2023; Wang et al., 2023).

In this study, we used the RoosterBio exosome-promoting system to prepare MSC-derived exosomes in the Hollow Fiber 3D bioreactor, analyzed subpopulations by harvesting exosomes at different stages to evaluate the heterogeneity differences of exosomes during long-term culture, and then detected the *in vivo* distribution of exosomes via tail vein administration of isotopically labeled exosomes in rats; subsequently, we tested the therapeutic effects of natural exosomes in a mouse silicosis model. The results of this study provide references for the stability of subpopulations during 28 days exosome preparation, the influence of purification processes on exosome activity, the effects of administration routes of natural exosomes on therapeutic efficacy, and the heterogeneity considerations for natural exosomes as raw materials for engineered exosome carriers.

## 2 Materials and methods

### 2.1 Cell source

Umbilical cord tissues were collected at the China-Japan Friendship Hospital. All donors gave informed consent, and the study was approved by the Ethics Review Board of the First Hospital of Jilin University (Approval No. 2023-676). Human umbilical cord-derived MSC (hUC-MSCs) were obtained from two healthy newborn umbilical cord tissue donors (26-year-old mother and 34-year-old mother). Donors were excluded based on the following criteria (chronic diseases such as diabetes, history of tumors or abnormal growth, genetic diseases, etc.). hUC-MSCs were established with protocol designed in Beijing Jizhongke Biotechnology Co., LTD. Which was based on the content of “*Isolation and Characterization of Mesenchymal Stromal Cells from Human Umbilical Cord and Fetal Placenta*” (Beeravolu et al., 2017), but the culture medium used was RoosterNourish™-MSC-CC (RoosterBio, K82016).

In brief, fresh umbilical cord tissue was aseptically collected, and the internal blood vessels were dissected using a scalpel to isolate the cord lining and Wharton’s Jelly. The tissues were then transferred to a separate dish, cut into 1–2 mm fragments with scissors, and placed into a new cell culture dish. A small volume of culture medium was added to facilitate tissue adhesion. Following a 1-h incubation at 37°C in 5% CO_2_, the medium was replaced with fresh culture medium. The medium was subsequently changed every 3 days. Between days 12 and 16, cells began to migrate from the tissue. At this point, the cells were enzymatically dissociated, collected, washed, and prepared as a single-cell suspension before being transferred to a new culture vessel for subculture. Quality testing included microbial testing, endotoxin testing, cell surface marker testing, and tri-lineage differentiation potential testing.

### 2.2 Static cell cultivation in 2D

The seed library comprised P2 generation umbilical cord MSCs, named hUC-MSC-0214 and hUC-MSC-1103, while the working library consisted of P4 generation cells. For experiments, P4 cells were expanded to P5. In brief, P4 hUC-MSCs, recovered from cryopreservation, were seeded at a density of 4 × 10^6^ cells in T-175 cell culture flasks (Thermo Fisher Scientific, 159910). After overnight adherence, the cells were washed twice with PBS, and the Rooster Nourish™-MSC-XF system (RoosterBio, K82016) was used for expansion culture, adding 5 mL of Rooster Booster™-MSC-XF additive to Rooster Basal™-MSC basal medium. The cells were cultured at 37°C with 5% CO_2_ for 4–5 days until reaching approximately 90% confluence. After harvesting, the cells were dissociated with TrypLE™ Select Enzyme (Thermo Fisher Scientific, 12563029), and the harvested cells were used for downstream experiments, including cell counting, activity testing, and culture supernatant centrifugation and filtration (using a 0.22 μm filter, Millipore, GSWP04700) for seeding cells in the Fiber Cell bioreactor. MSC expansion experiments were all conducted in T-175 culture flasks instead of Cell STACKs for ease of washing debris, maintaining cell viability, and precise control of cell numbers according to the number of culture flasks. Cell counting and cell viability were crucial for downstream long-term exosome production experiments.

### 2.3 Cell cultivation in fiber cell bioreactors

The Fiber Cell hollow fiber bioreactor system (FiberCell Systems, C2011), which has an internal circulating volume of 500 mL and an extra-capillary space (ECS) volume of 20 mL, was prepared according to the manufacturer’s instructions. The internal and external tubing of the reactor was pre-cleaned with PBS (Cytiva, SH30256.01) filtered through a 0.22 μm filter for 48 h at a flow rate of 30 mL/min, starting 4 days prior to cell seeding. On day 0, cells were seeded for the first time. After cell counting and viability testing, the supernatant was filtered through a 0.22 μm membrane, and the cells were suspended and seeded into the bioreactors. The seeding amount for hUC-MSC-0214 and hUC-MSC-1103 was 100 million cells each. According to the manufacturer’s recommendation, cells needed to stabilize in the reactor for 3 days, with flow rates set at 22 mL/min, 24 mL/min, and 26 mL/min for days 0–3, using 500 mL of MSC-XF complete medium externally. On day 3, the internal and external circulation was switched to 500 mL of Rooster Collect EV Pro™ medium (EV, RoosterBio, K41001) to start exosome production, with a flow rate set at 28 mL/min, increased to 30 mL/min on day 5. The second cell seeding was conducted on day 28 using the same method as above, with 200 million cells each for hUC-MSC-0214 and hUC-MSC-1103. From day 28 to day 30, the flow rate was maintained at 28 mL/min, and both internal and external circulation used MSC-XF complete medium. On day 30, the internal and external circulation environment was switched to 500 mL of MSC-EV medium to continue exosome production, with a flow rate of 30 mL/min.

### 2.4 Production of exosomes derived from MSCs

Starting from day 0, 2 mL of culture supernatant was withdrawn daily from the external circulation medium for glucose content and pH determination to monitor cell status. Every 2 days, 20 mL of culture supernatant was collected from the internal circulation, and 24 mL of preheated MSC-EV medium at 37°C was replaced. After the second cell seeding on day 28, the cells stabilized until day 30. From day 30 onwards, every 2 days, 20 mL of culture supernatant was collected, and 24 mL of preheated EV medium at 37°C was replaced. The collected supernatant was centrifuged at 300 *g* for 5 min and then at 3,000 *g* for 1 h. The supernatant was divided into aliquots of 18 mL, 1 mL, and 1 mL and stored at −80°C. The 18 mL aliquot was used for exosome purification, one 1 mL aliquot for Nanoparticle Tracking Analysis (NTA), and another 1 mL aliquot for Complete Extracellular Vesicle Characterization (ExoView) exosome subpopulation analysis.

### 2.5 Cell flow cytometry

For immunophenotypic analysis of hUC-MSCs, cells were harvested using Triple and prepared as single-cell suspensions through a 100 μm cell strainer (Corning, 352360). After counting, the cell concentration was adjusted to 5 × 10^6^/mL, and the cells were incubated with the corresponding antibodies from the BD Stemflow hMSC Analysis Kit (BD Bioscience, 562245) for 30 min at 4°C. After washing with PBS, cells were analyzed using a BD FACS Canto™ II flow cytometer, and data were analyzed using FlowJo (10.6.2) software.

### 2.6 Cytokine detection

Cell-free supernatants from UC-MSC-0214 under 2D MSC-XF and MSC-EV conditions were collected, centrifuged at 4°C and 900 × *g* for 10 min, and then stored at −80°C. Following the manufacturer’s recommendations, ELISA kits were used to determine the concentrations of FGF-2 (Abcam, ab99979), HGF (Abcam, ab275901), IL-8 (Abcam, ab214030), TIMP-1 (Abcam, ab187394), TIMP-2 (Abcam, ab270213), and VEGF-A (Abcam, ab289705) in pg/1E5 cells/day.

### 2.7 Cell tri-lineage differentiation

To assess the differentiation ability of hUC-MSCs, cells were induced in osteogenic (MesenCult™ Osteogenic Differentiation Kit, StemCell, 05465_C), adipogenic (MesenCult™ Adipogenic Differentiation Kit, StemCell, 05412_C), and chondrogenic (MesenCult™-ACF Chondrogenic Differentiation Kit, StemCell, 05455_C) differentiation media according to the manufacturer’s instructions. The media were changed at regular intervals. After differentiation, cells were stained with Alizarin Red for osteogenic cells, Oil Red O for adipocytes, and Alcian Blue for chondrocytes. Images of stained cells were obtained using an inverted microscope, and cell slice images were acquired using SlideViewer (3D Histech).

### 2.8 Exosome purification

#### 2.8.1 Exosome detection via the ultrafast-isolation system (exodus)

Cell culture supernatant stored at −80°C in 50 mL centrifuge tubes was thawed on ice, centrifuged at 4°C and 2,000 × *g* for 10 min, and transferred to new centrifuge tubes using a 20 mL syringe and 0.22 μm filter. Each 5 mL of supernatant was filtered using a 0.22 μm filter. The filtered supernatant was then transferred to the Exodus (HuiXinBio, H300) for fine purification.

#### 2.8.2 Tangential flow filtration (TFF)

Cell culture supernatants frozen at different time points were thawed on ice, and the initial samples were pre-filtered using a Sartopure GF (0.65 μm, 17.3 cm^2^) filter (Sartorius, 5555305 PS-FF-M). Clarified samples were then sterilized using a Sartopore 2XLG (0.8/0.2 μm, 17.3 cm^2^) filter (Sartorius, 5445307 GS-FF-M). Tangential flow filtration of the sample solution was performed using a Hydrosart 300 kDa 0.1 m^2^ ultrafiltration membrane pack (Sartorius, 3081447902E-SW). Throughout the filtration process, the flux for clarification filtration was 285 LMH with a pressure difference of 0–0.6 Bar, for sterilization filtration was 313 LMH with a pressure difference of 0–0.3 Bar, and for tangential flow filtration was a concentration of 2.6 times, with a 7-fold volume change of PBS, and an average filtration rate of 43 LMH, an inlet pressure of 0.4 Bar, a reflux pressure of 0 Bar, a transmembrane pressure of 0 Bar, and a TMP of 0.2 Bar. The harvested liquid was stored at 4°C for downstream fine purification.

#### 2.8.3 Monolithic chromatographic columns for exosome fine purification

Fine purification was performed using CIMmultus™ EV monolithic columns (Sartorius BIA Separations) in a binding-elution mode on an ÄKTA Avant (Cytiva). Two buffer solutions were used: equilibration buffer A (20 mM Tris, pH 8.03) and elution buffer B (20 mM Tris, 2 M NaCl, pH 8.03). After column installation, the column was washed with 20% ethanol using purified water for 10 column volumes (CVs), followed by cleaning with 1 M NaOH for 10 CVs, and then equilibration with buffer A. After stabilization of UV and conductivity, sample loading was initiated, and the elution process was monitored by UV values. After sample loading, the column was washed with buffer A for 10 CVs. During the elution process, a gradient was prepared using a combination of buffer A and buffer B, with a flow rate of 1 mL/min and a gradient of 0%–100% B over 30 min. Samples were collected based on the appearance of UV peaks on the equipment. After sample collection, the column was cleaned with 1 M NaOH, followed by preservation with 20% ethanol.

### 2.9 Exosome characterization

#### 2.9.1 Nanoparticle tracking analysis (NTA)

Nanoparticle tracking analysis (NTA) was performed to determine the size distribution and concentration of particles in the cell supernatant and purified exosomes. ZetaView PMX 110 (Particle Metrix, Meerbusch, Germany) and corresponding ZetaView 8.04.02 software were used to detect and analyze the size and concentration of particles in the cell supernatant and purified exosomes at each time point. Samples of separated cell supernatant or exosomes were appropriately diluted with 1 × PBS buffer for particle size and concentration measurement. NTA measurements were recorded and analyzed at 11 positions, with each sample recorded three times. The ZetaView system was calibrated using 110 nm polystyrene beads, with the temperature maintained at around 26°C.

#### 2.9.2 Transmission electron microscope (TEM)

Exosomes were visualized under a TEM using the recommended method for preparing exosome electron microscopy samples. In simple terms, 20 μL of exosome solution was fixed with 10 μL of 4% PFA (Beyotime, P0099). Then, 20 μL of the fixed sample was dropped onto Formvar/Carbon FCF300-Cu grids (Sigma-Aldrich, 930296) and stained with phosphotungstic acid solution for 10 min. After blotting with filter paper to remove excess liquid, the samples were air-dried at room temperature and imaged using a Hitachi HT7700 TEM.

#### 2.9.3 Wes protein simple (Wes)

Purified exosomes were analyzed using the Wes automated capillary-based immunoassay system (Bio-Techne) for typical exosome markers. Protein samples were loaded based on equal protein amounts, and data were analyzed using Compass software. Antibodies used included CD63 (Abcam, ab68418), CD81 (R&D Systems, MAB46152), CD9 (Cell Signaling Technology, 13174S), TSG101 (Millipore Sigma, T5826), Alix (Novus Biologicals, NBP1-49701), and Calnexin (Novus Biologicals, NB100-1965) as controls.

#### 2.9.4 ExoView

Exosome subpopulation detection was performed using the ExoView Tetraspanin chip (ExoView, Boston, MA, United States), which contains antibodies against CD81, CD63, and CD9 proteins. Mouse IgG1 was used as a negative control. 35 μL of the sample was dropped onto the chip surface and incubated overnight. After washing, the chip was treated with ExoView Tetraspanin Labeling ABs (EV-TC-AB-01), including CD9/Alexa 488, CD81/Alexa 555, and CD63/Alexa 647, for co-localization testing to characterize the subpopulations on the exosome surface. Imaging of the chip was performed using the ExoView R200 (ExoView) with single-particle interferometric reflectance imaging sensor (SP-IRIS) technology and ExoScan v0.998 (ExoView) acquisition software. Data analysis was performed using ExoViewer 3.2, with a size threshold set from 50 to 200 nm in diameter.

#### 2.9.5 Tunable resistive pulse sensing (TRPS)

The concentration, size distribution, and diameter of purified exosomes were measured using the TRPS technique (qNano IZON system; Izon, Cambridge, MA, United States). The system was calibrated for voltage, stretch, pressure, and baseline current using two standard beads: CPC100B (mode diameter: 114 nm, concentration: 1.0 × 10^13^/mL) and CPC70D (mode diameter: 70 nm, concentration: 3.0 × 10^13^/mL). Detection was performed using 40 μL of diluted samples and an NP100 nanopore (suitable for a size range of 50–200 nm), with data analysis conducted using Izon Data Suite (1.0.2) software.

### 2.10 Exosome activity assay

*In vitro* scratch assays were performed using Human Dermal Fibroblast Cells (HSF) and Human Umbilical Vein Endothelial Cells (HUVEC). Cells were seeded at a density of 5 × 10^5^ cells/well in 6-well plates (Thermo Fisher Scientific, 140675). After cell monolayer formation, scratches were made using a sterile 200 μL pipette tip with three lines spaced 0.5 cm apart. After washing with PBS, exosomes at different concentrations were added, with PBS as the control. The scratch area was observed under a microscope at 0, 12, and 24 h, and cell migration area analysis was performed using ImageJ software. For the angiogenesis assay, HUVECs were seeded at a density of 1 × 10^5^ cells/well in Matrigel-coated 24-well plates (Corning, 354248). Exosomes at different concentrations were added, with PBS as the control, and cultured for 24 h. Three fields were randomly selected at 12 h and 24 h, and endothelial cells were counted and analyzed for the number of blood vessels formed using ImageJ software.

### 2.11 ^89^Zr-labeled exosome

Biodistribution Exosome solution was exchanged with buffer containing NaHCO_3_ using a Sephadex G-25 PD10 column (Cytiva, 17085101), and desferrioxamine (DFO) was mixed with exosomes at a ratio of 1:10 by weight. The mixture was incubated at 37°C for 1 h on a constant temperature shaker, followed by purification using a PD10 column again. After NaCl elution, ^89^Zr-DFO-Exosomes were obtained. ^89^Zr-labeled exosomes were slowly injected into the rat tail vein, and whole-body small animal PET scans were performed at 1 h, 2 h, 6 h, 1 day, 2 days, 5 days, and 7 days post-administration. Each bed was scanned for 10 min. The scanning window was set at 350–650 Kev. After completion of small animal PET/CT scanning, images were reconstructed, and data were processed using PMOD software. Regions of interest were delineated for the brain, heart, lungs, liver, spleen, kidneys, knee joints, and muscles. The radioactivity concentration (i.e., radioactivity value per unit volume) of the regions of interest was obtained, and the decay-corrected activity at each time point was calculated. The percentage of injected dose per gram of tissue (%ID/g) was calculated based on the administered dose and the radioactivity concentration.

### 2.12 Silicosis mouse model and ethical statement

Seventy-two male SPF C57BL/6J mice, aged 6–8 weeks, weighing 18–24 *g*, were purchased from Vital River Laboratory Animal Technology Co., Ltd. The C57BL/6J mice were randomly divided into six groups, with 12 mice per group. Six mice per cage were housed in polycarbonate/polypropylene cages within an IVC system, with *ad libitum* access to food and water throughout the experimental period. The environmental conditions were set at a temperature of 20°C–26°C, relative humidity of 40%–70%, and a 12-h light-dark cycle. The mice were divided into the following groups: normal control group, silica model group, MSC intravenous administration group, MSC pulmonary administration group, exosome intravenous administration group, and exosome pulmonary administration group. All the animal experiments were performed in China-Japan Friendship Hospital Animal Center (Approval No. ZRDWLL230141) under sterile condition in an SPF facility in accordance with the animal welfare laws and the regulations of the Association for Assessment and Accreditation of Laboratory Animal Care (AAALAC).

An experimental silicosis model in mice was induced by intratracheal instillation of silica suspension. Silica particles were heated at 200°C for 2 h to inactivate potential contaminating endotoxins and suspended in sterile saline at a concentration of 50 mg/mL. The suspension was sonicated for 10 min before use. Mice in the model and treatment groups were anesthetized with isoflurane and intratracheally instilled with 100 μL (50 mg/mL) of silica suspension, at a dose of 5 mg/mouse. The cell therapy group received cell therapy 1 day after modeling, with a cell dose of 5 × 10^5^/mouse, administered once. The exosome therapy group received exosome therapy 1 day after modeling, with exosome particles at a dose of 4 × 10^9^ particles/mouse, administered 5 times with a single dose interval of 4 days.

The experiment was terminated 21 days after modeling. Mice were euthanized, and organs were collected. The lungs were excised, fixed in formalin-zinc solution for 24 h, embedded in paraffin, sectioned (5 µm) using TSRI’S Histology Core, and stained with hematoxylin and eosin (H&E) and Masson’s trichrome stain. Paraffin-embedded sections were scanned onto glass slides and stored in Digital Image Hub. Lung injury was evaluated in a blinded manner by scoring the percentage of lung lobes affected by alveolitis (maximum score of 200), as well as peribronchitis and perivasculitis (maximum score of 200) in four right lung lobes and a single left lung lobe. The total lung score (TLS) (maximum score of 500) was determined by combining the assessment data of alveolitis, peribronchitis, and perivasculitis.

### 2.13 Statistical methods

The data are presented as mean ± SD, with the number of experiments and mice (n) detailed in the figure legends. Data collection and normalization were conducted using Microsoft Excel. Statistical differences between two groups were assessed using an unpaired Mann–Whitney U-test with a two-tailed distribution, performed in Prism 9 software (GraphPad Software). All statistical analyses were conducted with a significance threshold set at *p* < 0.05.

## 3 Results

### 3.1 MSCs-EV medium enhances exosome production without impairing MSC function

Achieving large-scale production of exosomes involves two strategies: scaling up cell culture platforms to increase total yield and enhancing exosome yield per individual cell. Previous reports suggested issues with cell differentiation in adipose-derived stromal cells during long-term culture in Fiber Cell bioreactors. Therefore, we initially tested the changes in cell morphology, phenotype, and function of MSCs during 50 days exosome production under MSC-EV culture conditions in 2D cell culture flasks, as well as the differences in exosomes relative to MSC-XF.

**FIGURE 1:**
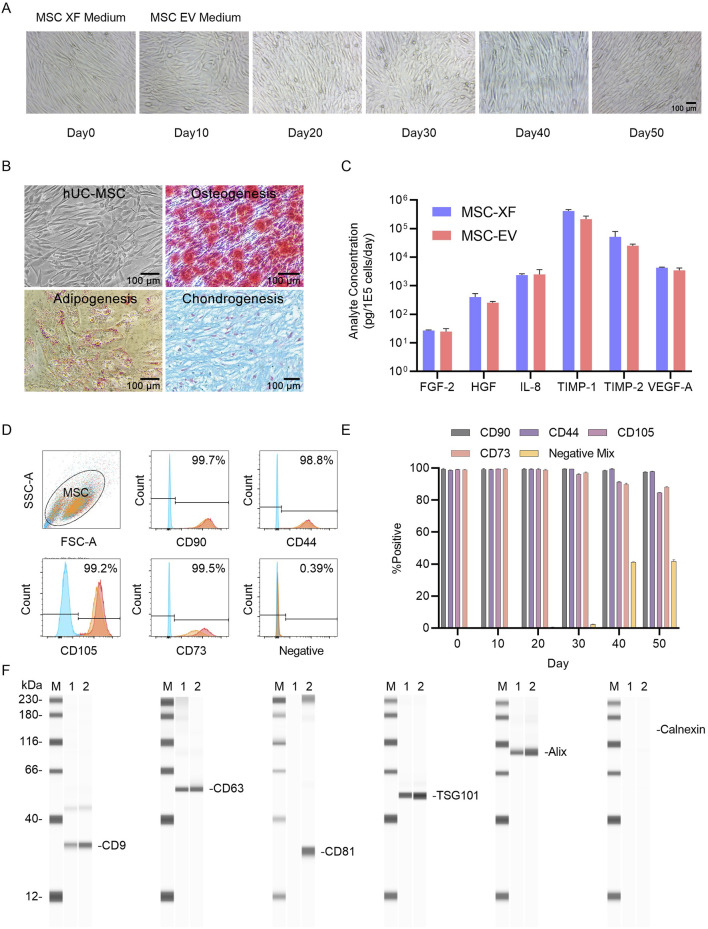
2D Functional assessment of umbilical cord MSCs (UC-MSCs) transitioning from expansion culture to exosome production. **(A)** Microscopic images of hUC-MSC-0214 cells in MSC-XF expansion medium and at different time points in MSC-EV culture medium. MSC-XF Day0 represents the time point when cells reached 85% confluence in the 2D culture environment, followed by a switch to MSC-EV culture medium. **(B)** Evaluation of mesodermal differentiation capability of hUC-MSC-0214 after 20 days in MSC-EV culture medium. Representative microscopic images of hUC-MSCs (1), chemical staining to detect adipocytes (2), osteocytes (3), and chondrocytes (4) after inducing differentiation into three mesodermal lineages. Adipocytes were stained with Oil Red O, osteocytes with Alizarin Red, and chondrocytes with Alcian Blue. **(C)** Comparison of secretion of angiogenesis-related cytokines by hUC-MSC-0214 under MSC-XF (Day 0) and MSC-EV (Day 20) culture conditions. **(D)** Flow cytometric analysis of surface markers on MSCs under different culture conditions. Blue indicates cells stained with IgG isotype control, red represents cells stained with corresponding antibodies under MSC-XF (Day 0) conditions, and orange represents cells stained with corresponding antibodies under MSC-EV (Day 20) conditions to detect positive and negative markers. “Negative” indicates staining with a cocktail of antibodies for detection of MSC-negative markers including CD34, CD11b, CD19, CD45, and HLA-DR. **(E)** Flow cytometric analysis of surface markers on MSCs under MSC-EV culture conditions at different culture times. **(F)** Detection of exosome marker proteins in cell supernatant and nanoparticle-enriched fluid after purification by Exodus using Wes Protein Simple. “M” denotes Marker, “1” represents nanoparticle-enriched fluid after purification of MSC-XF supernatant by Exodus, “2” represents nanoparticle-enriched fluid after purification of MSC-EV supernatant by Exodus. Exosome-specific positively expressed proteins include CD9, CD63, CD81, TSG101, and Alix, while Calnexin is a negative exosomal protein marker.

Firstly, MSCs exhibited a typical spindle-shaped structure under MSC-XF culture conditions. Upon switching to MSC-EV culture conditions, cells showed an increase in transverse diameter, along with the appearance of more vesicular structures within the cells (Figure 1A). During this period, we collected MSCs cultured under MSC-EV conditions for 30 days and conducted differentiation ability tests, revealing that MSCs still retained the potential for osteogenic, adipogenic, and chondrogenic differentiation (Figure 1B). Additionally, we examined the secretion of hematopoietic cytokines under MSC-XF (Day 0) and MSC-EV (Day 30) conditions, finding no significant differences between them (Figure 1C), indicating that MSC functionality was not affected under MSC-XF and MSC-EV culture conditions.

MSCs collected at different time points were subjected to flow cytometry analysis, revealing no significant changes in cell phenotype under MSC-XF culture conditions for 4 days and MSC-EV culture conditions for 30 days (Figure 1D). However, after 40 days of culture, the expression levels of CD105 and CD73 decreased, while the expression level of Negative Mix increased, indicating a certain degree of differentiation of MSCs during long-term culture under MSC-EV conditions (Figure 1E).

Furthermore, we collected cell culture supernatants from MSC-XF culture for 4 days and MSC-EV culture for 30 days, filtered them through a 0.22 μm membrane, purified them using Exodus, and quantified the protein content using BCA protein assay. We then detected the expression of exosome-specific proteins post-purification. Following Exodus purification, exosome-specific markers CD9, CD63, TSG101, and Alix were detected in both MSC-XF and MSC-EV culture supernatants, indicating enrichment of exosomes in the culture supernatant. By comparing the expression levels of Alix and the non-specific exosome marker Calnexin, it was determined that MSC-EV culture supernatants had a higher exosome content compared to MSC-XF culture supernatants under equal number of exosomes loading conditions. Moreover, based on the expression level of CD81, it was inferred that exosome subpopulations harvested under different culture conditions exhibited differences (Figure 1F).

These results demonstrate that under 2D conditions, MSCs expanded under MSC-XF conditions and then switched to MSC-EV culture conditions for exosome production, within 30 days, the secretion of hematopoietic cytokines by MSCs was not significantly affected, and the cells retained tri-lineage differentiation capability. There were no changes in cell phenotype within 30 days. Following purification by Exodus under equal protein loading conditions, exosomes enriched in the culture supernatant of MSC-EV culture accounted for a higher proportion than those in the supernatant of MSC-XF culture, and the production of CD81^+^ exosomes indicating a richer subpopulation of exosomes harvested from MSC-EV.

### 3.2 Hollow fiber bioreactor sustains 28 days MSC exosome production

**FIGURE 2:**
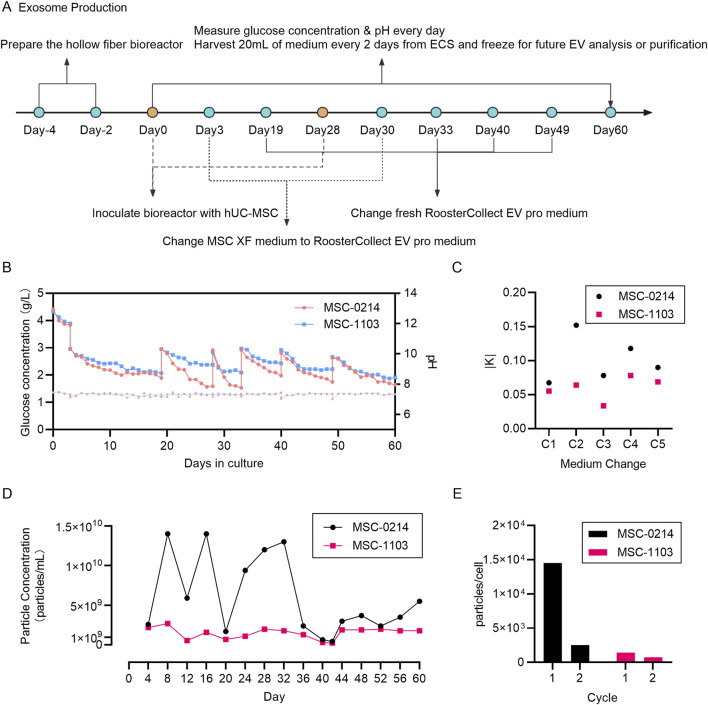
Monitoring of culture parameters during exosome production in a hollow fiber bioreactor demonstrates the feasibility and consistency of maintaining cellular homeostasis for long-term exosome preparation. **(A)** Schematic diagram illustrating the entire workflow of EV production by hUC-MSCs in the hollow fiber bioreactor system. The hollow fiber cell culture system requires a 4-day preparation period, followed by cell seeding, wherein PBS circulates for 2 days, followed by complete cell culture medium MSC-XF circulation for 2 days. On Day 0, hUC-MSCs are seeded into the hollow fiber cartridges. After a 3-day cell seeding period, MSC-EV culture medium replaces the inner and outer circulating MSC-XF medium. From Day 4 to Day 28, a 25-day EV production period begins, with 2 mL of culture medium extracted daily from the external circulation for monitoring glucose concentration and pH. Every 2 days, 20 mL of EV-rich cell-conditioned medium is collected from the internal circulation and frozen for future EV isolation and analysis. On Day 28, new parent hUC-MSCs are seeded into their respective hollow fiber cartridges, with both inner and outer circulating media being MSC-XF, and on Day 30, the circulating media are changed to MSC-EV culture medium. From Day 31 to Day 60, a 30-day EV production period begins, with monitoring and sample collection conducted in the same manner as in the first production cycle. **(B)** Daily monitoring of glucose concentration in the circulating culture medium to ensure continuous glucose consumption by the cells. Two data points per day represent glucose supplementation by adding fresh cell culture medium. pH measurement using circulating culture medium samples ensures that cells do not undergo metabolic stress. Red represents hUC-MSC-0214 cells, and blue represents hUC-MSC-1103 cells. **(C)** Comparison of glucose consumption rates before and after media change. Black represents hUC-MSC-0214 cells and red represents hUC-MSC-1103 cells. **(D)** Nanoparticle concentration of EV-rich fluid harvested from the internal circulation at different time points. Black represents hUC-MSC-0214 cells and red represents hUC-MSC-1103 cells. **(E)** Number of particles secreted per single cell harvested during different production cycles. Black represents hUC-MSC-0214 cells and red represents hUC-MSC-1103 cells.

Hollow fiber cell scaffolds provide cells with continuous nutrition over time through the structure of semi-permeable fibers, enabling continuous exosome production over several weeks. Simultaneously, metabolites are transferred into the internal circulation to enrich extracellular space exosomes, which increases both the total yield of exosomes and the concentration of exosomes per unit volume. Additionally, cells in the Hollow Fiber environment are in a proliferative inhibition state, ensuring minimal changes in cell number within the bioreactor, thus promoting the stability of subpopulations during long-term exosome preparation. We first hypothesized that different parent cell lines exhibit varying exosome release capacities. To test this, we compared exosome production over 30 days under identical conditions using two parent cell lines. Additionally, we explored whether continuous cell culture supplementation affects exosome production and subpopulation distribution. Therefore, on the 28th day, we supplemented with a higher number of cells and continued cultivation until the 60th day (Figure 2A). Particle concentrations in the supernatant were measured using NTA, and heterogeneity of exosome subpopulations was assessed using Exoview.

According to the glucose consumption curve, 0214 cells exhibited a higher glucose consumption rate compared to 1103 cells over both experimental periods. The absolute value of the slope of glucose consumption before and after each medium change (K0214) was greater than that of K1103, and pH curves remained stable (Figures 2B,C).

During the initial production cycle (CYCLE 1, Days 4–28), we observed significant differences in extracellular vesicle output between cell types. 0214 cells demonstrated higher productivity with a supernatant concentration of 9.08 × 10^9^ particles/mL (1.45 × 10^4^ particles/cell), while 1103 cells showed substantially lower secretion at 1.58 × 10^9^ particles/mL (0.25 × 10^4^ particles/cell). This indicates that under the same culture conditions, exosome production is influenced by the inherent secretion capacity of parent cells. Following reactor reseeding and extended culture (CYCLE 2, Days 31–60), both cell types exhibited reduced productivity though with distinct patterns. 0214 cells experienced a dramatic 70.26% reduction in total particle yield (2.70 × 10^9^ particles/mL) and a 90.34% decrease in per-cell secretion (0.14 × 10^4^ particles/cell). In contrast, 1103 cells maintained relatively stable production with only 10.76% fewer total particles (1.41 × 10^9^ particles/mL) and a 42.86% reduction in per-cell output (0.08 × 10^4^ particles/cell) compared to CYCLE 1 (Figures 2D,E).

These results demonstrate that the Fiber Cell bioreactor and MSC-EV exosome production system can achieve 28 days exosome production, influenced by both parent cell type and the number of seeded cells. Assuming that cells cultured for the initial 30 days are consistent with the efficiency of re-seeded cells (particle counts in supernatant on Day 28 remaining at median levels), higher cell density on fibers results in lower exosome production per cell. It is important to note that the final exosome yield is not solely based on cell release but also involves the process of cell re-capturing exosomes. Thus, optimizing cell seeding quantity, exosome harvesting time, and medium replacement intervals are necessary to achieve a balance between exosome yield and preparation cycle. Moreover, stability of glucose consumption and pH alone cannot assess the cellular status during the exosome secretion phase.

### 3.3 Hollow fiber bioreactor with EV medium preserves stability of principal subpopulations

**FIGURE 3:**
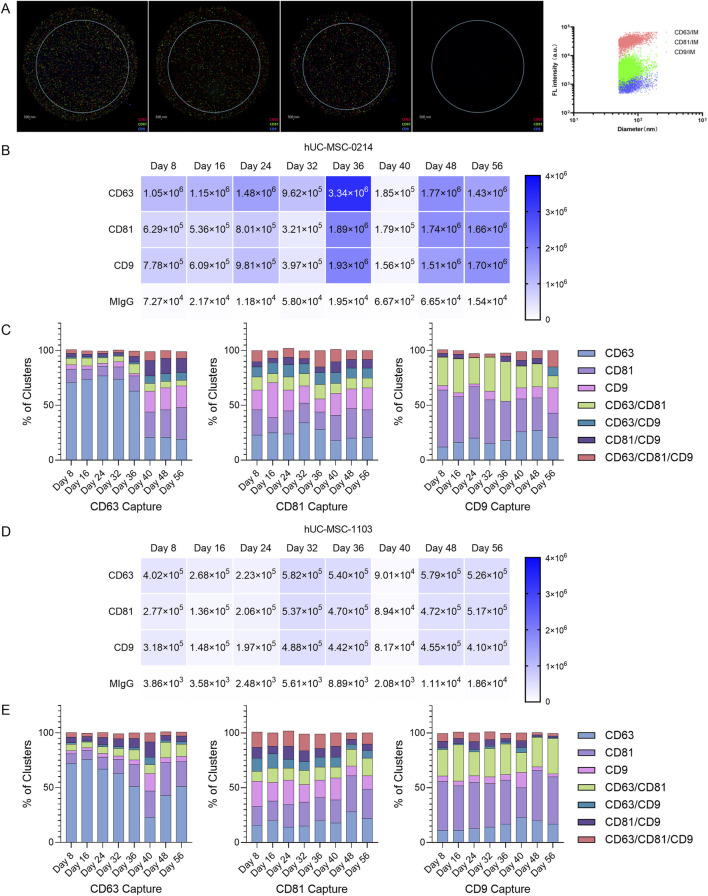
Evaluation of the stability of exosome subpopulations’ composition during the production cycle using NanoView chips. **(A)** Enrichment of nanoparticle samples from hUC-MSC-0214 (Day 10) based on NTA particle concentration data for ExoView detection. Images of exosomes captured in CD63, CD81, CD9, and negative control capture channels, where red capture points represent CD63^+^, green capture points represent CD81^+^, and blue capture points represent CD9^+^. **(B)** Dilution of hUC-MSC-0421 samples at different time points based on NTA particle concentration data to the ExoView detection range, showing actual captured exosome counts in each channel of the chip. **(C)** Radar plot analysis of relative co-expression levels of CD63/CD81/CD9 multi-markers in hUC-MSC-0214 samples at different time points. **(D)** Dilution of hUC-MSC-1103 samples at different time points based on NTA particle concentration data to the ExoView detection range, showing actual captured exosome counts in each channel of the chip. **(E)** Radar plot analysis of relative co-expression levels of CD63/CD81/CD9 multi-markers in hUC-MSC-1103 samples at different time points.

Exosomes are highly multifunctional vesicles, and those released by cells exhibit heterogeneity, wherein different subpopulations of exosomes display distinct compositions and/or functions. Identification and isolation of exosome subpopulations are crucial for understanding exosome biology and function. Furthermore, stability of harvested exosome subpopulations is an essential quality control standard for long-term exosome production processes. Therefore, using Single Particle Interferometric Reflectance Imaging Sensing (SP-IRIS) technology and antibody-labeled probes, we captured and analyzed the composition of major exosome subpopulations harvested at different time points from initial solutions from 0214 to 1103 cells (Figure 3A).

Initially, based on particle concentration results from NTA, and following supplier recommendations, we adjusted the particle concentration of harvested solutions from different time points to 1 × 10^8^ particles/mL using PBS filtered through a 0.22 μm membrane. Exosomes were captured on chips labeled with CD9/CD81/CD63 antibodies. This adjusted particle concentration ensured more accurate image analysis data, with exosome concentrations captured in each channel falling within the optimal range for data analysis. The total number of exosome particles captured by each channel from 0214 cells (Figure 3B) was higher than that from 1103 cells (Figure 3D), indicating the presence of more non-exosome particles in the solution from 1103 cells.

A change in exosome subpopulation stability was defined as an extreme difference in the percentage range (R = max-min) of any subpopulation exceeding 10% during the experimental period. Any adjacent data difference (Max|Xn-Xn+1|) appearing in absolute values was considered a change in subpopulations at a specific time point.

Based on fluorescence particle capture counts, differentiation in 0214 sample subpopulations occurred on Day 36 in the CD63 capture channel, with changes in subpopulations observed in CD63 (R = 58.24%), CD81 (R = 19.95%), CD9 (R = 18.07%), and CD81/CD9 (R = 12.34%) (Figure 3C). Specifically, between Day 8 and Day 36, the median particle percentage for CD63 was 73.84%, for CD81 was 10.53%, for CD9 was 19.96%, and for CD81/CD9 was 2.76%. However, between Day 40 and Day 56, the median particle percentage for CD63 decreased to 20.72%, for CD9 to 3.23%, while it increased for CD81% to 24.64%,and for CD81/CD9 to 13.90%.

In the CD81 capture channel, subpopulation changes occurred in CD63 (R = 16.07%), CD81 (R = 12.79%), and CD9 (R = 20.08%). Between Day 8 and Day 36, the median particle percentage for CD63 was 24.62%, for CD81 was 18.02%, and for CD9 was 17.76%. Conversely, between Day 40 and Day 56, the median particle percentage for CD63 decreased to 20.30%, while it increased for CD81% to 24.78%, and for CD9 to 19.55%.

In the CD9 capture channel, subpopulation changes occurred in CD63 (R = 14.93%), CD81 (R = 29.52%), CD9 (R = 21.28%), and CD63/CD81 (R = 25.45%). Between Day 8 and Day 36, the median particle percentage for CD63 was 15.55%, for CD81 was 41.80%, for CD9 was 3.58%, and for CD63/CD81 was 30.64%. Between Day 40 and Day 56, the median particle percentage for CD63 increased to 26.30%, and for CD9 was 10.04%, while it decreased for CD81 was 29.91% and for CD63/CD81 was 20.31%.

According to the fluorescence particle capture counts, subpopulation differentiation in 1103 samples occurred on Day 36 in the CD63 capture channel, with changes observed in CD63 (R = 51.17%), CD81 (R = 21.32%), and CD9 (R = 13.36%) (Figure 3E). Between Day 8 and Day 36, the median particle percentage for CD63 was 66.63%, for CD81 was 10.94%, and for CD9 was 2.91%. Conversely, between Day 40 and Day 56, the median particle percentage for CD63 decreased to 42.52%, while it increased for CD81% to 24.45%, and for CD9 to 4.67%.

In the CD81 capture channel for 1103 samples, subpopulation changes occurred in CD63 (R = 14.26%), CD81 (R = 15.59%), and CD9 (R = 13.41%). Between Day 8 and Day 36, the median particle percentage for CD63 was 15.59%, for CD81 was 20.55%, and for CD9 was 16.81%. Between Day 40 and Day 56, the median particle percentage for CD63 was 21.99%, for CD81 was 27.40%, and for CD9 was 12.48%.

In the CD9 capture channel, subpopulation changes occurred in CD63 (R = 12.31%), CD81 (R = 19.24%), CD9 (R = 11.05%), and CD63/CD81 (R = 14.38%). Between Day 8 and Day 36, the median particle percentage for CD63 was 12.60%, for CD81 was 41.22%, for CD9 was 5.15%, and for CD63/CD81 was 26.06%. Between Day 40 and Day 56, the median particle percentage for CD63 increased to 19.76%, for CD81 was 43.32%, and for CD63/CD81 was 26.63%, while it decreased for CD9 was 3.08%.

These results indicate that the composition of exosome subpopulations harvested between Day 8 and Day 36 remained stable. It is noteworthy that the maximum R value appeared in the CD63 subpopulation of the CD63 channel, the CD9 subpopulation of the CD81 channel, and the CD81 subpopulation of the CD9 channel for 0214, and the CD63 subpopulation of the CD63 channel, the CD81 subpopulation of the CD81 channel, and the CD81 subpopulation of the CD9 channel for 1103. The same maximum subpopulation change group was observed in CD63 Capture-CD63 and CD9 Capture-CD81, suggesting that despite different parent cells, similar changes occurred in the two groups of exosome subpopulations. These changes may result from either long-term effect of Cycle 1 cells (alterations in cell phenotype after continuous 2D cultivation for 40 days) or the introduction of Cycle 2 cells (the impact of newly introduced cells on the internal environment, or the effects of changing XF expansion medium during the period). But, after subpopulation changes occurred on Day 36, the composition remained stable.

### 3.4 The combination separation technique sustains the exosome activity

In the quality monitoring of large-scale exosome production, challenges arise due to the limited volume of culture supernatant that can be harvested at different time points and the processing time required for liquid. Therefore, it is essential to establish a method for the rapid purification of exosomes to facilitate their separation and downstream characterization. Additionally, exosomes purified using this method should not exhibit changes in their physical properties or biological functions compared to those obtained via large-scale purification techniques. In this study, tangential flow filtration combined with strong anion exchange chromatography was employed as a purification process for large-scale exosome production, while Exodus was used for the purification of small-volume exosomes at different time points during exosomal preparation.

**FIGURE 4:**
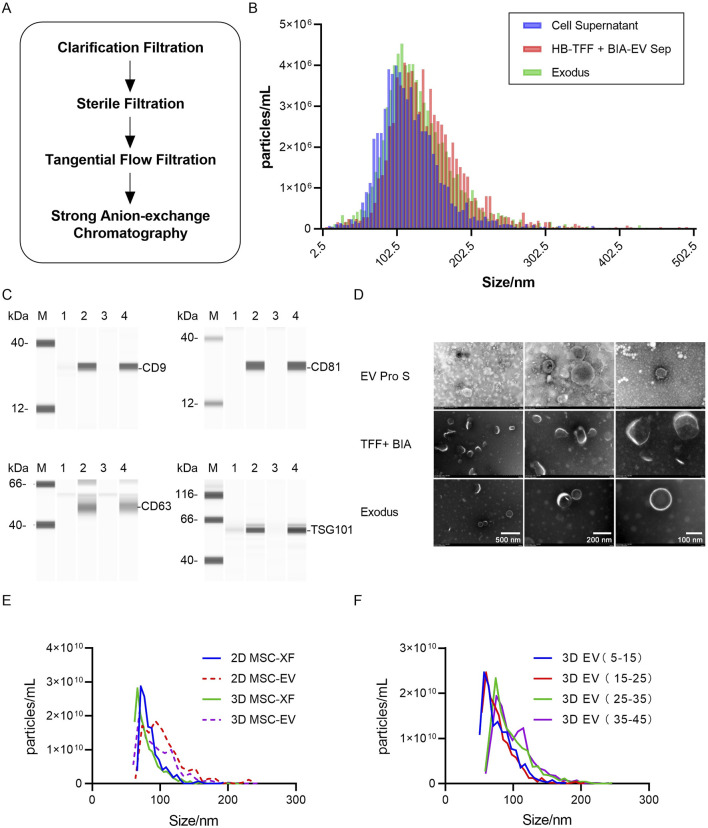
Fine separation of exosomes achieved using tangential flow filtration combined with strong anion exchange chromatography. **(A)** Schematic diagram illustrating the process of fine separation of exosomes, with clarification using a 0.65 μm filter, sterilization using a 0.8/0.2 μm filter, tangential flow filtration using a 300 kDa membrane, and strong anion exchange chromatography column using CIMmultus™ EV. **(B)** Particle size distribution of exosome concentrates harvested from different exosome separation methods as detected by TRPS. Blue represents the original solution of mixed exosomes from Hollow Fiber hUC-MSC-0214 Day 4–40 before separation, red represents exosome concentrates after tangential flow filtration combined with strong anion exchange chromatography, and green represents nanoparticle concentrates after enrichment by Exodus. **(C)** Detection of exosome marker proteins in nanoparticle-enriched fluid after separation by TFF + BIA Strong AEX and Exodus using Wes Protein Simple. “M” denotes Marker, “1” represents mixed original sample 1 of hUC-MSC-0214, “2” represents exosome-enriched fluid after separation by TFF + BIA-EV, “3” represents mixed original sample 2 of hUC-MSC-0214, and “4” represents nanoparticle-enriched fluid after separation by Exodus. Exosome-specific positively expressed proteins include CD9, CD63, CD81, and TSG101. **(D)** Electron microscopy images showing typical exosome structures of enriched particles in the mixed original sample of hUC-MSC-0214, exosome-enriched fluid after separation by TFF + BIA-EV, and nanoparticle-enriched fluid after separation by Exodus at resolutions of 500 nm, 200 nm, and 100 nm. **(E)** Particle size distribution of exosomes harvested from hUC-MSC-0214 under different culture media and conditions, with all detected samples being nanoparticle-enriched fluid after Exodus enrichment. “2D XF” represents a mixture of cell supernatants from MSC-XF culture Day 1–3, “2D EV” represents a mixture of cell supernatants from Day 4 to 10, “3D XF” represents a mixture of cell supernatants from Hollow Fiber bioreactor Day 1–3, and “3D EV” represents a mixture of cell supernatants from Day 4 to 40. **(F)** Particle size distribution of exosomes harvested from hUC-MSC-0214 at different time stages in the Hollow Fiber bioreactor, with all detected samples being nanoparticle-enriched fluid after Exodus enrichment. “3D EV 5–15” represents a mixture of cell supernatants from Day 5 to 15, “3D EV 15–25” represents a mixture of cell supernatants from Day 15 to 25, “3D EV 25–35” represents a mixture of cell supernatants from Day 25 to 35, and “3D EV 35–45” represents a mixture of cell supernatants from Day 35 to 45.

The combination separation method, utilizing TFF in conjunction with BIA EV Separation, has been tested for its ability to purify exosomes while maintaining their activity and achieving high recovery efficiency (Figure 4A). Initially, a mixture of samples from Days 4 to 40, consisting of 200 mL of huMSC-0214 3D MSC-EV harvested supernatant, was used. However, due to limitations in sample volume for this purification method, the sample volume harvested was insufficient to meet the requirements for testing the particle size differences of exosomes at different time points. Consequently, we chose to purify 0214 exosome with Exodus in the same mixed sample as that used for TFF + BIA EV Strong anion exchange (AEX) purification, as well as samples of exosomes harvested at different time points.

Firstly, differences in particle size distribution of exosomes harvested at different time points and under different culture conditions were analyzed using TRPS. Subsequently, the physical properties of exosomes harvested by the two purification methods were compared based on differences in protein expression and electron microscopy images.

According to TRPS particle size and concentration results, the particle size distribution of the supernatant from cell culture, after passing through a 0.22 μm filter, was consistent with that after purification by Exodus, with median (X50) values of 113.5 nm and 114.7 nm, respectively. However, after TFF + Strong AEX purification, the main peak of the particle size distribution shifted slightly to the right, indicating slightly larger particle sizes, with a median (X50) value of 119.6 nm (Figure 4B). This suggests that Exodus purification, due to its non-exosome-specific purification method, results in a particle size distribution closer to that of the supernatant. Both purification methods yielded particles with significant expression of exosome-specific proteins, indicating that both methods achieved enrichment of exosomes in the supernatant (Figure 4C). Combining these results with electron microscopy images, particles obtained by both purification methods exhibited typical cup-shaped structures of exosomes (Figure 4D).

Comparing the concentration and particle size distribution of exosomes harvested from 2D XF (Days 1–3), 2D EV (Days 4–10), 3D XF (Days 1–3), and different stages of 3D EV Pro (Days 4–40) supernatant after Exodus purification, the mean particle size for 2D XF was 86 nm (SD = 17.4), for 2D EV was 108 nm (SD = 43.1), for 3D XF was 82 nm (SD = 22.4), and for 3D EV was 95 nm (SD = 27.7). The particle size distribution of exosomes harvested under the same culture conditions in 2D and 3D environments was similar, with exosomes harvested in the 3D environment being smaller than those harvested in the 2D environment. Furthermore, the mean particle size for 3D exosomes harvested from Days 5–15 was 78 nm (SD = 22.4), from Days 15–25 was 78 nm (SD = 22.5), from Days 25–35 was 97 nm (SD = 29.6), and from Days 35–45 was 101 nm (SD = 35.2) (Figures 4E,F). Exosomes produced during the first cycle from Days 5–25 did not exhibit a shift in particle size, but upon the introduction of new cells for the second cycle, from Days 25–45, there was an increase in particle size, particularly in the range of 100–140 nm, while particles in the range of 60–100 nm decreased.

In summary, exosome particle size changes with different purification methods, culture conditions, and culture media. Exosomes harvested in a 3D environment have smaller particle sizes compared to those harvested in a 2D environment. During the 3D culture of MSC-EV, exosome production from Days 5–25 exhibited stable particle sizes under the same environmental and culture media conditions. However, upon the addition of new cells, from Days 25–45, the particle size of exosomes increased over time, with particle size directly correlating with subpopulation composition (ExoView fluorescence vs. particle size). Both culture media and environmental conditions influence the composition of exosome subpopulations, with stable subpopulation composition observed under the same conditions. Any changes in either factor (bioreactor, culture media, or internal cells) will affect the composition of exosome subpopulations.

**FIGURE 5:**
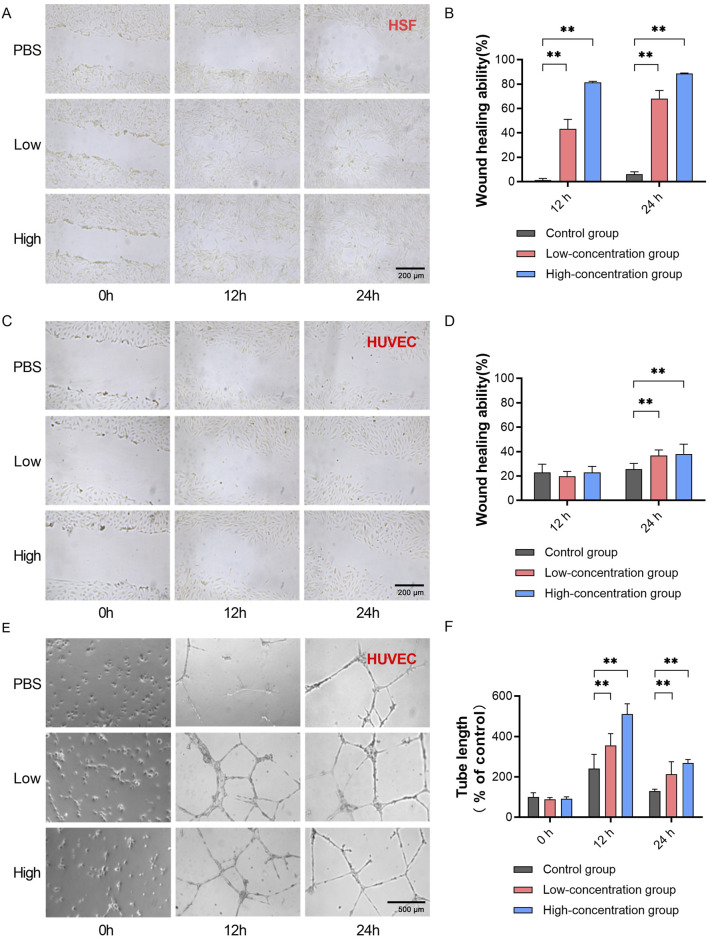
Exosomes separated by TFF + BIA-EV exhibit activity in promoting wound healing and angiogenesis *in vitro*. **(A)** Wound healing assay using HSF cells treated with exosomes at different concentrations, measuring HSF migration ability (magnification ×100). **(B)** Quantitative analysis of HSF migration ability at different time points under the influence of exosomes at various concentrations, *N* = 6. **(C)** Wound healing assay using HUVEC cells treated with exosomes at different concentrations, measuring HUVEC migration ability (magnification ×100). **(D)** Quantitative analysis of HUVEC migration ability at different time points under the influence of exosomes at various concentrations, *N* = 6. **(E)** *In vitro* matrix tube formation assay evaluating angiogenic capacity of HUVECs after treatment with exosomes at different concentrations (magnification ×100). **(F)** Quantitative analysis of the length of newly formed blood vessels in HUVECs at different time points under the influence of exosomes at various concentrations, *N* = 12. The control group was used as the standard. The error bars represent the standard error of the mean of six measurements (**p ≤ 0.01).

We subsequently tested the biological activity of EVs purified by TFF + Strong AEX through scratch assays and tube formation assays using HUVECs and HSF cells. EVs were tested at two concentrations: 2 × 10^8^ particles/mL and 4 × 10^8^ particles/mL. In the scratch assays with HSF cells, both concentrations of EVs showed enhanced cell migration compared to the control group (Figure 5A). The healing distances at 12 h and 24 h were greater in the EV-treated groups. At 12 h, the healing rates were significantly higher in the low-dose group (43.28%, SD = 7.79) and high-dose group (81.42%, SD = 2.15) compared to the control (1.25%, SD = 1.36). At 24 h, the healing rates were also significantly higher in the low-dose group (68.09%, SD = 6.63) and high-dose group (88.68%, SD = 0.84) compared to the control (6.11%, SD = 1.92) (Figure 5B). In the HUVEC scratch assays, after 24 h of treatment with EVs at both concentrations, there was a moderate enhancement in cell migration, with greater healing distances compared to the control group (Figure 5C). At 24 h, the healing rates were significantly higher in the low-dose group (36.69%, SD = 4.63) and high-dose group (38.03%, SD = 7.98) compared to the control (25.54%, SD = 4.75) (Figure 5D). In the HUVEC tube formation assays, after 12 h of treatment with EVs at both concentrations, there was a significant enhancement in tube formation capability. At 24 h, although the tube formation capability started to decline in all groups compared to the control, it remained enhanced in both the low-dose and high-dose groups (Figure 5E). Compared to the PBS control at 12 h, the length of new blood vessels was notably higher in the low-dose group (355.70%, SD = 58.51) and high-dose group (511.18%, SD = 51.00). At 24 h, the length of new blood vessels was also significantly higher in the low-dose group (213.33%, SD = 61.81) and high-dose group (268.17%, SD = 18.67) compared to the control (91.40%, SD = 10.24) (Figure 5F). These results demonstrate the significant biological activity of EVs purified by TFF and BIA Strong AEX, promoting cell migration in HSF and HUVEC cells, as well as angiogenesis in HUVECs.

### 3.5 Tissue-specific distribution of exosomes upon intravenous injection into rat tail veins

We proceeded to investigate the tissue-specific distribution of exosomes following intravenous injection into rat tail veins. This exploration is crucial for advancing the development of exosome-based therapeutic biologics, as it allows for the determination of the exogenous exosome’s biodistribution and pharmacokinetic characteristics in animal models. Tracking exosomes *in vivo* provides vital insights into their biodistribution, migratory capabilities, toxicity, biological effects, communication abilities, and mechanisms of action. While many exosome imaging techniques label the lipid bilayer of exosomes with various imaging dyes, these methods may track phospholipids taken up by cells rather than the exosomes themselves. Compared to fluorescent or bioluminescent imaging, radiolabeling offers several advantages *in vivo* tracking of biomolecular therapies due to its excellent sensitivity for deep tissue imaging and potential for quantitative measurements.

**FIGURE 6:**
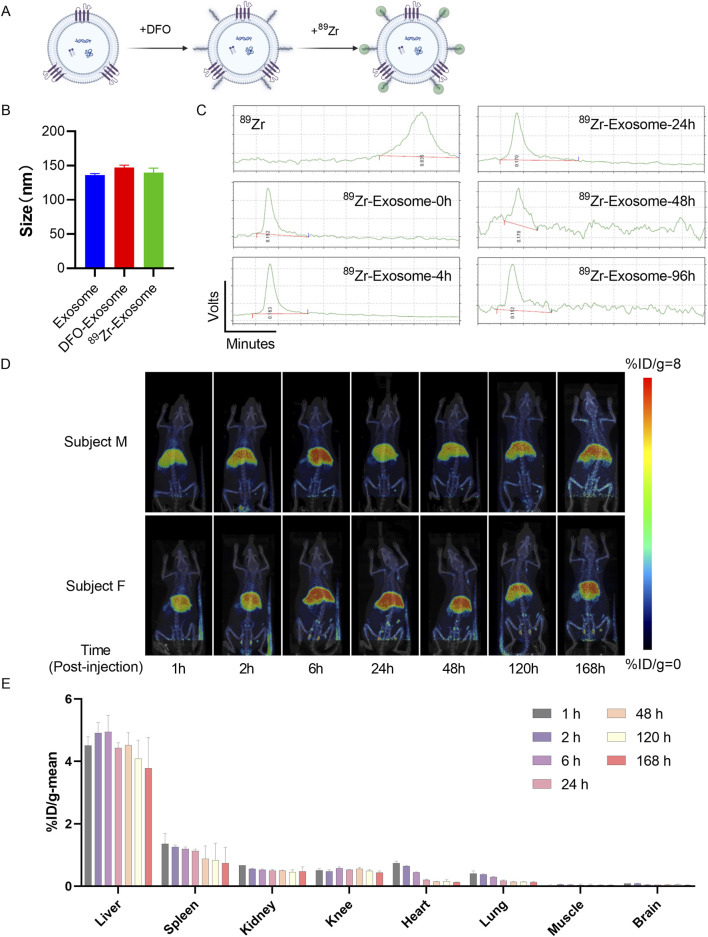
Assessment of *in vivo* biodistribution of ^89^Zr-labeled exosomes in rats using PET/CT imaging. **(A)** Schematic diagram illustrating the method of surface labeling of exosomes with ^89^Zr. **(B)** Particle size distribution of ^89^Zr-labeled exosomes detected by NTA, presented as mean ± standard deviation. **(C)** Radioactive stability of ^89^Zr-labeled exosomes within 96 h. **(D)** Evaluation of the biodistribution of ^89^Zr-Exosomes in rats using PET/CT imaging, represented as %ID/g at each time point. **(E)** Analysis of the overall distribution of ^89^Zr-Exosomes in various organs of rats (*N* = 4) using PET/CT images, represented as %ID at different time points in the regions of interest.

Initially, we engineered ^89^Zr-DFO-Exosomes [DFO: desferrioxamine] (Figure 6A). Nanoparticle tracking analysis (NTA) revealed consistent particle size distributions for initial exosomes, DFO-exosomes, and ^89^Zr -DFO-Exosomes, with X50 values of 136.27 nm, 147.23 nm, and 139.9 nm, respectively (Figure 6B), indicating that this labeling method did not significantly affect the particle size of exosomes. Subsequent quality control of the labeled product, ^89^Zr -DFO-Exosomes, was performed using Radio-instant thin-layer chromatography (Radio-iTLC), with zirconium oxalate (^89^Zr) solution serving as the control and 0.5 M citric acid/sodium citrate buffer as the developing agent. Thin-layer scanner detection and calculation of Rf values confirmed that the radiochemical purity (RCP) of ^89^Zr -DFO-Exosomes was >90%. Using the BCA method, we determined the protein concentration of ^89^Zr -DFO-Exosomes and calculated their radioactivity, specific activity, and vesicle concentration. Each rat was administered a radioactive dose of approximately 100 μCi, with the ratio of exosomes to ^89^Zr -DFO-Exosomes adjusted to achieve a vesicle-specific activity of 5.5E-12 mCi/Particles, resulting in an exosome dose of 7.5 × 10^10^ Particles/kg.

Samples of ^89^Zr-DFO-Exosomes were stored in physiological saline at 4°C, and Radio-iTLC was used to monitor the radiochemical purity (RCP) at different time points, demonstrating that the RCP of ^89^Zr -DFO-Exosomes remained >90% over 96 h (Figure 6C). With the experimental administration time set at 4 h, the stability of radioactive detection of ^89^Zr -DFO-Exosomes during the experiment was confirmed. Subsequently, we evaluated the *in vivo* distribution of ^89^Zr labeled exosomes in a rat model following intravenous administration via tail vein injection. Whole-body small animal PET scans were performed at 1, 2, 6, 24, 48, 120, and 168 h post-administration. In SD rats, following a single intravenous injection of ^89^Zr labeled exosomes, the majority of the radioactive material was distributed in the liver, followed by the spleen, kidneys, knee joints, heart, and lungs, with lower distribution observed in muscle and brain tissues (Figure 6D). The radioactive uptake values in the spleen, heart, kidneys, and brain peaked at 1 h post-administration, with average values of 1.36%ID/g, 0.74%ID/g, 0.67%ID/g, 0.41%ID/g, and 0.09%ID/g, respectively. The radioactive uptake in muscle peaked at 2 h post-administration, with an average value of 0.05%ID/g. The radioactive uptake in the liver and knee joints peaked at 6 h post-administration, with average values of 4.95%ID/g and 0.58%ID/g, respectively. By 168 h post-administration, all tissues showed varying degrees of decline in radioactive uptake, with the heart exhibiting the greatest decrease, reaching 18% of its peak value; the lungs, spleen, and brain tissues decreased to 30%–55% of their peak values; and muscle, kidneys, and liver decreased to 70%–80% of their peak values (Figure 6E).

These results demonstrate that exogenous exosomes, when administered via the tail vein in rats, primarily accumulate in liver tissue. For indications targeting specific tissues, consideration should be given to the route of administration and the modification of exosome membranes to achieve tissue-targeting functionality.

### 3.6 Respiratory tract exosome administration improves lung function in silicosis mouse model

**FIGURE 7:**
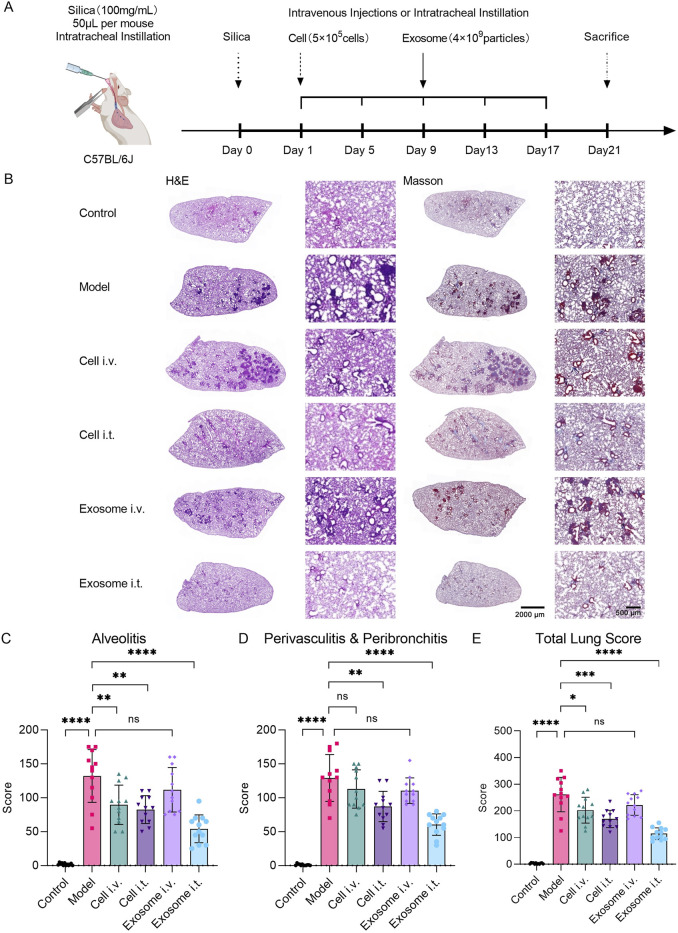
Respiratory exosome administration alleviates silica-induced lung injury in mice. **(A)** Schematic diagram illustrating the experimental design of exosome therapy in a silica-induced silicosis mouse model. **(B)** Evaluation of lung tissue pathological changes using H&E and Masson staining. Images demonstrate pathological alterations in lung tissue induced by silica, with alleviation of lung injury upon respiratory exosome administration. **(C)** Study of pulmonary pathological indicators in mice induced by silica, with re-evaluation of alveolar inflammation scores in lung sections stained with H&E after different treatment regimens (*N* = 12). **(D)** Study of pulmonary pathological indicators in mice induced by silica, with re-evaluation of peribronchitis and perivasculitis scores in lung sections stained with H&E after different treatment regimens (*N* = 12). **(E)** Study of pulmonary pathological indicators in mice induced by silica, with re-evaluation of total lung scores in lung sections stained with H&E after different treatment regimens (*N* = 12). The model group was used as the standard. The error bars represent the standard error of the mean of twelve measurements (****p ≤ 0.001, ***p ≤ 0.001, **p ≤ 0.01, *p ≤ 0.05, and ns > 0.05).

Previous studies have reported the therapeutic effects of 3D bioreactor-cultured umbilical cord MSC-derived exosomes administered via tail vein injection in an experimental silicosis model induced in C57BL/6J mice. To assess the *in vivo* biological function of exosomes, we conducted a 21-day experiment using a similar silicosis animal model. In addition to the groups treated with cells and exosomes via tail vein, based on the distribution of exosomes *in vivo*, we added groups receiving respiratory tract administration of cells and exosomes to assess the effects of different administration routes on therapeutic outcomes in the silicosis model. Based on previous studies, respiratory administration of exosomes effectively targets the lungs (Dinh et al., 2020). Therefore, the tissue distribution studies have opted for caudal vein injection as the delivery method (Figure 7A).

H&E and Masson staining results depicted the degree of pulmonary interstitial fibrosis, cellular nodules, and lung blue collagen deposition at the end of the experiment. Compared to the normal control group, the silica model group exhibited a significant increase in lung fibrosis. However, both the airway cell-treated group and airway exosome-treated group showed significant improvement in fibrosis severity and collagen deposition symptoms, whereas no significant improvement was observed in the tail vein cell-treated group and tail vein exosome-treated group (Figure 7B).

The lung total score was determined by integrating the percentage score for pulmonary inflammation in four right lung lobes and one left lung lobe (Figure 7C), along with scores for peribronchitis and perivasculitis (Figure 7D). In the inflammation score, the mean score for the normal control group was 1.83 (SD = 1.85), for the model group was 132.08 (SD = 38.99), for the tail vein cell-treated group was 89.58 (SD = 29.03), for the airway cell-treated group was 82.50 (SD = 20.64), for the tail vein exosome-treated group was 111.67 (SD = 32.84), and for the airway exosome-treated group was 54.17 (SD = 20.54) (Figure 7C). In the peribronchitis and perivasculitis score, the mean score for the normal control group was 1.01 (SD = 1.28), for the model group was 129.17 (SD = 34.50), for the tail vein cell-treated group was 112.92 (SD = 28.24), for the airway cell-treated group was 87.08 (SD = 22.31), for the tail vein exosome-treated group was 110.42 (SD = 18.88), and for the airway exosome-treated group was 60.42 (SD = 15.73) (Figure 7D). In the lung total score, the mean score for the normal control group was 2.83 (SD = 2.37), for the model group was 261.25 (SD = 64.46), for the tail vein cell-treated group was 202.50 (SD = 48.41), for the airway cell-treated group was 169.58 (SD = 32.30), for the tail vein exosome-treated group was 222.08 (SD = 38.40), and for the airway exosome-treated group was 114.58 (SD = 22.10) (Figure 7E).

Based on the lung total score results, it is believed that in the silica-induced silicosis mouse model, single intravenous administration of cells, respiratory tract cell administration, and respiratory tract exosome administration can all significantly improve lung injury, with respiratory tract exosome administration being superior to the other two groups. However, tail vein exosome administration did not show significant therapeutic effects. This might be because tail vein cell administration can still maintain a certain number of cells trapped in the lungs, while after tail vein exosome administration, the lungs are not the main distribution site for exosomes.

These results indicate that respiratory tract exosome administration in the silica-induced silicosis mouse model designed in this study can achieve better improvement in lung injury than tail vein cell administration and respiratory tract cell administration. MSC-derived exosomes can serve as an alternative to MSCs in the treatment of silicosis in mice.

## 4 Discussion

Exosomes, as a novel and efficient cell-free therapy, hold great promise but face significant challenges in large-scale production, particularly in reducing costs. To address these issues, expanding cell culture platforms and utilizing 3D bioreactors can enhance exosome yields (Grangier et al., 2021). Hollow fiber bioreactors, for instance, provide an increased surface area for cell attachment, allowing for a higher cell density and greater exosome production. In these systems, MSCs attach to semi-permeable fibers, with nutrients and gases supplied through the medium, while waste is expelled into the extracellular space. A prime example of this is the Fiber Cell C2011, which can support up to 1E9 cells on a 4,000 cm^2^ surface. However, microcarriers with smaller surface areas may impact cell nutrition. In this hollow fiber bioreactor, 5E8 adipose stem cells (ASCs) are capable of producing between 1E12 and 3E12 exosome particles over a period of 4–7 weeks, corresponding to 12–14 mg of proteins (Cadwell et al., 2016). In contrast to microcarrier stir-tank reactors, where continuous cell proliferation can lead to loss of key subpopulations such as CD81, cells in hollow fiber reactors are typically in a proliferation-suppressed state. Furthermore, environmental factors like hypoxia or small molecule stimulation can further boost exosome secretion (Yang et al., 2022; Bavafa et al., 2025). RoosterBio’s EV culture media, for example, have been shown to improve single-cell exosome yields, shortening production time and reducing costs (Gobin et al., 2021).

While closed automated bioreactors and GMP-compliant culture media (e.g., RoosterBio’s EV media) address yield limitations, the inherent heterogeneity of MSCs and their secreted exosomes poses a critical challenge to clinical translation. MSCs derived from diverse tissue sources, such as bone marrow, umbilical cord, or adipose tissue, exhibit distinct differentiation potentials, cytokine secretion profiles, and metabolic states, which are mirrored in the molecular composition and functional properties of their exosomes. Even within a single tissue source, donor variability, passage number, and culture conditions (e.g., hypoxia or inflammatory priming) further amplify cellular heterogeneity, leading to inconsistencies in exosome yield, subpopulation distribution, and therapeutic efficacy. For instance, our study revealed that umbilical cord-derived MSCs cultured in 3D bioreactors exhibited donor-dependent variations in exosome secretion rates (Figure 2). Kowal et al. (2016) demonstrated tetraspanin-defined exosome subpopulations (e.g., CD63^+^, CD81^+^) exhibit distinct molecular cargo and functional specialization. Similarly, Willms et al. (2016) highlighted that exosome subpopulations from the same MSC source can mediate divergent biological effects. Therefore, exosome subpopulation stability during the long-term production of exosomes is critical for therapeutic consistency.

Exosome subpopulation variability underscores the need for stringent process standardization, including the use of multi-omics characterization to identify functional biomarkers and engineered purification platforms for isolating therapeutically active exosome subpopulations. Within the EV Pro technical framework, our multidimensional analysis using ExoView and TRPS demonstrated relative compositional stability of exosome subpopulations over 28 days (Figure 3), suggesting potential functional consistency during this temporal window. Notably, the ExoView platform demonstrated distinct advantages in enabling direct *in situ* analysis without requiring sample purification procedures.

Beyond biological variability, scalable isolation methods remain a bottleneck. As exosomes are being developed for large-scale biomedical and clinical applications, there is an increasing demand for rapid, reliable, high-yield, and high-purity separation methods (Dilsiz, 2024). Common techniques include differential ultracentrifugation (DUC) and density gradient ultracentrifugation (DGUC). While DUC is widely used, it is time-consuming, unsuitable for large volumes, and can result in contamination by non-exosomal proteins. DGUC offers improved efficiency and purity and remains the gold standard for laboratory-scale isolation, but still suffers from particle aggregation and contamination issues (Li et al., 2018; Duong et al., 2019). In addition, its scalability limitations render it impractical for industrial biomanufacturing, particularly when staggered harvesting intervals and small-volume optimization are required during early process development. Alternatively, immunoaffinity methods separate exosomes based on antigen-antibody specificity, enabling the isolation of specific subpopulations. However, these methods are often expensive, time-consuming, and require strict conditions (Mondal and Whiteside, 2021). Another approach, polymer precipitation (e.g., using PEG), can effectively precipitate exosomes by reducing solubility and is suitable for large volumes. However, it may lead to contamination with lipoproteins or viral particles (Rider et al., 2016).

Ultrafiltration, a size-based separation technique, uses filtration membranes with varying pore sizes or molecular weight cutoffs (MWCO) to remove impurities. Larger contaminants are blocked while smaller components pass through. However, while ultrafiltration is cost-effective, it often results in lower exosome purity and may compress the particles during filtration. This method is divided into dead-end filtration (DEF), which works well for small volumes but leads to rapid filter cake buildup, and TFF, which prevents clogging and improves exosome yield (Kim et al., 2021; Putra et al., 2023). Size-exclusion chromatography (SEC) is another technique that separates exosomes based on size, with larger particles eluting faster. SEC not only preserves exosome integrity but can also be enhanced with strong anion exchanger for higher purity. When combined, ultrafiltration and SEC can yield higher purity and quantities of exosomes, providing a cost-effective solution for large-scale production (Bellotti et al., 2021; Silva et al., 2023). Currently, the cost-effective large-scale exosome isolation processes primarily rely on TFF coupled with SEC, as well as TFF combined with strong AEX size-exclusion monolithic columns. These integrated methodologies represent the prevailing industrial-scale approaches capable of yielding high-purity exosomes. Our hybrid TFF + Strong AEX strategy effectively balances three critical metrics for clinical translation: yield, purity, and functional consistency. To ensure quality control during scalable process development, we rigorously validated the consistency of this approach against the Exodus ultrafast-isolation system for small-volume samples. Comparative analyses confirmed comparable particle size distributions, marker expression profiles, and typical exosome structures between the two methods, with these validation results systematically presented in Figures 4B–D.

Crucially, therapeutic success depends not only on production consistency but also on delivery route optimization. Our findings diverged from previous studies, as we observed that airway exosome administration significantly improved disease progression in a mouse silicosis model, while intravenous delivery did not show similar effects (Hao et al., 2024). This highlights the importance of the delivery route in achieving therapeutic outcomes, as different methods affect pharmacokinetics, bioavailability, and overall drug efficacy (Zhou et al., 2020). Therefore, choosing the optimal delivery route based on the exosome’s properties and therapeutic goals is crucial for protecting the payload, minimizing degradation, extending circulation time, and enhancing both efficacy and safety.

Intravenous administration remains the most common route for exosome delivery. Once injected, exosomes circulate through the blood, penetrate tissues, and accumulate in specific target areas. Most exosomes circulate alongside red blood cells and are cleared by the liver and spleen, with only a small fraction interacting with white blood cells and endothelial cells. The particle size of exosomes can influence their distribution-smaller particles tend to enter liver sinusoids, while larger particles are cleared by the spleen. Macrophages in the liver and spleen primarily take up exosomes, and inhibiting macrophage activity can extend their plasma half-life. The uptake of exosomes by other organs depends on factors like concentration and circulation time (Wu et al., 2022). Moreover, genetic engineering of exosomes can allow targeting to tumor antigens, improving tumor tissue permeability and enhancing therapeutic effects, making them particularly useful in cancer therapy (Shi et al., 2025).

In contrast, nebulized inhalation represents an innovative, non-invasive delivery method for exosomes. Exosomes’ lipid membrane vesicles are stable, pressure-resistant, and small enough to be absorbed through the pulmonary epithelium, making them ideal candidates for nebulization. Once nebulized, exosomes form aerosolized particles that are absorbed via the pulmonary mucosa, allowing for targeted lung delivery. This method not only increases local drug concentrations but also reduces systemic side effects, making it particularly useful for lung diseases (Chu et al., 2022). Nebulized exosomes have shown promise in treating respiratory conditions such as bronchitis, laryngitis, and pneumonia, and in COVID-19, they can suppress lung immune storms and aid in lung repair. By combining COVID-19 virus RBD with lung-derived exosome vaccines, RBD retention in the lungs is enhanced, inflammation is reduced, and allergic reactions are avoided (Wang et al., 2022). Moreover, lung-derived exosomes exhibit higher bioavailability of mRNA and proteins in the lungs compared to other nanoplatforms, such as liposomes (Dinh et al., 2020).

Intranasal administration also represents a promising delivery route for exosomes, owing to their nanoscale lipid vesicle structure, which facilitates easy absorption through the nasal mucosa. The extensive absorption surface and rich submucosal blood vessels in the nasal cavity allow for rapid drug uptake. Notably, exosomes can enter the cerebrospinal fluid via the olfactory and trigeminal nerve pathways, bypassing the first-pass effect and avoiding the clearance mechanisms associated with oral or intravenous administration (Pandey et al., 2022; Giovannelli et al., 2023). This makes nasal exosome delivery particularly valuable for treating neurological disorders. For instance, exosomes loaded with PTEN and small interfering RNA have promoted axon growth and motor coordination in spinal cord injury rats, while exosomes containing curcumin have reduced α-synuclein aggregation and improved neuronal function in Parkinson’s disease mice (Offen et al., 2019; Mobahat et al., 2023). These promising results underscore the potential of intranasal exosome delivery in the treatment of neurological diseases.

Natural MSC-derived exosomes deliver bioactive factors such as lipids, proteins, and miRNA to exert biological functions, demonstrating significant potential in treating and preventing lung diseases (Raghav and Mann, 2024). However, challenges such as limited targeting, variable active factor loading, and *in vivo* clearance still exist. To address these, targeting peptides are fused with exosomal membrane proteins to create targeted exosomes capable of delivering disease-specific drugs (Tian et al., 2023; Shi et al., 2025). For instance, exosomes overexpressing CD24, combined with damage-associated molecular patterns (DAMPs), inhibit NF-κB pathway-mediated inflammation and have shown safety and efficacy in mouse models of lung diseases like sepsis, allergic asthma, COPD, and pulmonary fibrosis (Tsioulos et al., 2022; Grigoropoulos et al., 2024). Additionally, engineered exosomes can be developed by fusing the RVG peptide with the LAMP2B scaffold protein. These LAMP2B-RVG exosomes, loaded with miRNA-124, can cross the blood-brain barrier, target ischemic cortical areas, and promote neurogenesis (Yang et al., 2017). Studies have also shown that exosomes expressing integrins α6β4 and α6β1 bind to lung fibroblasts and epithelial cells, influencing lung metastasis, while exosomes with integrin αvβ5 specifically bind to Kupffer cells, facilitating liver metastasis (Hoshino et al., 2015). Thus, enhancing exosome targeting to specific organs remains a promising area for further research.

Despite these challenges, MSC-derived exosomes demonstrate compelling advantages in biocompatibility and allogeneic safety, with emerging clinical evidence supporting their therapeutic potential in drug delivery and regenerative applications. Future success hinges on maintaining subpopulation stability during scaled production while advancing functional validation across preclinical and clinical models.

## Data Availability

The original contributions presented in the study are included in the article/supplementary material, further inquiries can be directed to the corresponding authors.
